# Female gender and autism: underdiagnosis and misdiagnosis – clinical and scientific urgency

**DOI:** 10.3389/fpsyt.2025.1704579

**Published:** 2026-01-05

**Authors:** Roberta Minutoli, Chiara Marraffa, Chiara Failla, Giovanni Pioggia, Flavia Marino

**Affiliations:** 1 Institute for Biomedical Research and Innovation (IRIB), National Research Council of Italy (CNR), Messina, Italy; 2 Faculty of Psychology, International Telematic University Uninettuno, Roma, Italy; 3 Department of Cognitive, Psychological Science and Cultural Studies, University of Messina, Messina, Italy

**Keywords:** autism, camouflaging, diagnosis, female, female autistic phenotype

## Introduction

Autism Spectrum Disorder (ASD) is a neurodevelopmental condition characterized by difficulties in communication and social interaction, along with restricted interests and repetitive behaviors. Symptom expression is highly heterogeneous, encompassing a wide range of functional impairments and levels of severity (1, 2). Epidemiological data indicate a higher prevalence of ASD among males than females. Some studies suggest that this apparent gender disparity is largely attributable to underdiagnosis and/or misdiagnosis in females, rather than a true lower incidence of the condition (3, 4). Population-based screening studies have estimated a true prevalence ratio of approximately 3.25 males for every female (5), and population-based predictive models suggest that up to 39% more girls could be expected to be diagnosed with ASD than are currently identified (6). Research indicates that cognitively competent females with ASD tend to be diagnosed significantly later than their male peers, despite similar levels of parental concern and a comparable number of professional referrals (7, 8). Females are more likely to receive subthreshold or alternative diagnoses, such as pervasive developmental disorder not otherwise specified or social communication disorder, rather than a full diagnosis of ASD (9, 10). Several studies have reported that females must exhibit greater intellectual or behavioral difficulties (11), emotional difficulties (12), or more pronounced ASD features (13) than males to receive a diagnosis, while parent-reported repetitive or restricted behaviors tend to lead to ASD diagnoses more frequently in males (12). Historically, the conceptualization and nosology of ASD have been shaped by predominantly male clinical samples (14, 15). The term “female” is primarily used to refer to people assigned female at birth (AFAB). However, we recognize that gender identity is distinct from biological sex, and that some transgender or non-binary individuals may share characteristics of the female autistic phenotype. Therefore, when we discuss autistic women and girls, we intend to include both AFAB individuals and people who identify as female, acknowledging the variability of experience and identity. Consequently, current diagnostic criteria and assessment tools are largely based on a male-centered understanding of autism, potentially overlooking the distinctive features of a female autistic phenotype (FAP). If such a sex/gender-specific phenotype exists, current diagnostic frameworks may fail to adequately capture it (16, 17), resulting in many autistic women remaining under the diagnostic radar. Clinical observations and autobiographical accounts further suggest that many girls and women on the autism spectrum engage in camouflaging or masking behaviors – compensatory social strategies aimed at hiding autistic traits – such as deliberately adapting facial expressions or tone of voice to conform to social norms, often practicing these behaviors in front of a mirror (18). These early-learned adaptive behaviors make autistic traits less recognizable to professionals and contribute to delayed or missed diagnoses (19, 37). However, the effort required to maintain such camouflaging often comes at a considerable emotional and psychological cost, increasing vulnerability to anxiety, depression, and burnout among autistic women (18). Taken together, these findings underscore the need to more clearly define and operationalize a Female Autism Phenotype (FAP). Future work should compare and adapt existing diagnostic instruments by integrating gender-sensitive probes and explicit measures of camouflaging. A coordinated research and clinical roadmap—with short-, medium-, and long-term objectives—will be essential to improving the identification, diagnosis, and support of autistic women across the lifespan. In this our opinion paper, we translate “camouflaging” as “masking,” meaning the set of behavioral and cognitive strategies that autistic people use to adapt to social norms and hide autistic traits. “Camouflaging” is considered a subcategory of masking, specifically referring to the imitation or reproduction of social behaviors observed in others, without altering internal experience.

## Female autism: a different phenotype

We can distinguish two main perspectives on female autism can be distinguished. On one side, the FAP is conceptualized as a relatively stable set of clinical characteristics, including patterns of interests that appear socially acceptable, relational styles that are superficially coherent, and internalized repetitive behaviors, which differ in frequency or manifestation from those typically observed in males (21). Qualitative studies have shown that these features contribute to delayed diagnosis in women (21). On the other side, the camouflaging perspective interprets many observed aspects in females as the result of adaptive processes and compensatory strategies—such as masking, scripting, and imitation—implemented to reduce the visibility of autistic traits (22). Quantitative research, for example using the CAT-Q, indicates that females with autism score higher on overall camouflaging compared to males (22, 37). These perspectives are not mutually exclusive: an integrated view suggests that FAP encompasses both genuine phenotypic differences and outcomes of camouflaging processes that alter the clinical presentation. Accordingly, research and clinical practice should simultaneously assess observable features (interest patterns, relational and repetitive behaviors) and subjective compensatory processes (masking, scripting, social exhaustion). For instance, a woman may display socially acceptable interests while relying on pre-learned scripts for peer interactions, reflecting both FAP and camouflaging. This integration highlights that delayed diagnoses and misattribution of comorbidities in women may result not only from different phenotypic expressions but also from camouflaging mechanisms that obscure the detection of autism (23, 24). Diagnostic criteria for ASD are primarily based on male samples, which contributes to missed or delayed diagnoses in females (see **Table 1**). The FAP is characterized by features that diverge from typical male presentations, particularly in the social-relational domain, demonstrating interests and social skills that are perceived by others as socially appropriate and well-developed (23–26). During initial social exposure, neurodivergent girls and women are often labeled as shy or reserved due to their more withdrawn behavior. Many women with FAP demonstrate advanced skills in some forms of nonverbal communication, such as gestures or facial expressions, which may mask autistic traits. However, some challenges persist in social interaction, such as interpreting sarcasm, metaphors, or implicit intentions, which depend more on contextual understanding than on basic nonverbal skills. Separating these domains helps clarify which difficulties are genuine phenomena of the female phenotype and which reflect compensatory or masking strategies. One distinctive feature of FAP is an enhanced capacity for observation, which manifests as a tendency to internalize social rules before actively engaging in interactions, despite frequent experiences of loneliness and frustration in forming and maintaining friendships (27, 28). In this context, camouflaging becomes a central aspect of FAP, characterized by the voluntary implementation of emotional and behavioral strategies to align with social expectations, thereby masking core autistic traits (29, 30). Camouflaging has several implications for psychological well-being, often resulting in emotional crises, psychosomatic symptoms (31), loss of spontaneity, identity confusion, chronic fatigue, and an increased risk of anxiety and depression (32, 33).

**Table 1:** Diagnostic tools: gender biases, supporting evidence, and proposed adaptations.

Diagnostic tool	Gender bias/limitation	Recent evidence	Proposed adaptation or alternative
ADOS-2 (Autism Diagnostic Observation Schedule)	Items largely derived from male samples; reduced sensitivity to camouflaging and subtle social reciprocity in females	lower sensitivity for female participants (41)	Add clinical probes for compensatory strategies and social tone; complement with self-report tools such as CAT-Q
ADI-R (Autism Diagnostic Interview – Revised)	Emphasis on early observable behaviors; limited assessment of relational development	bias toward externalized symptoms (29)	Incorporate qualitative parental narratives and contextual developmental histories
RAADS-R (Ritvo Autism Asperger Diagnostic Scale – Revised)	Focus on “atypical” interests and overt repetitive behaviors	possible under-identification in females (20)	Revise items to include socially normative but restricted interests (e.g., animals, fashion, relationships)
CAT-Q (Camouflaging Autistic Traits Questionnaire)	New measure; focuses on internal compensatory processes	validated self-report for camouflaging (22)	Systematically include in adult diagnostic protocols as complementary assessment
GQ-ASC (Gender Quotient Autism Spectrum Checklist)	Specifically designed for gender-sensitive autism screening	initial validation studies (42)	Encourage cross-cultural validation and integration in clinical practice

## Diagnostic biases and current instrument limitations

Standard diagnostic tools, such as the Autism Diagnostic Observation Schedule – Second Edition (ADOS-2) (34) and the Autism Diagnostic Interview-Revised (ADI-R) (15), may have reduced sensitivity for identifying autism in females. Women with autism spectrum disorder (ASD) often exhibit internalizing, restricted, or repetitive behaviors, or employ compensatory strategies such as masking, social scripting, and mimicry, which are less frequently captured by standard items (21–23, 35). Measurement invariance studies reveal sex-related differences in item factor loadings across several ADOS-2 modules, suggesting that some items perform differently for women and that targeted probes or item revisions may be necessary (23, 36). Moreover, camouflage strategies can lead to under-identification or delayed diagnosis in girls, particularly when behaviors appear culturally acceptable, such as restricted interests in animals, dolls, or singers, or when social difficulties are attributed to shyness or anxiety rather than neurodivergence (3, 4, 37). Many female-specific autistic manifestations are subtle or internalized, with nonverbal communication often being the most affected component, including rigid postures, difficulty interpreting sarcasm, or challenges understanding metaphors. Test administration may be further complicated by literal interpretations or narrow responses, as observed in measures such as the RAADS-R, requiring contextual examples for accurate assessment (38). During adolescence, emotional dysregulation or cognitive inflexibility may be misclassified within other diagnostic frameworks, leading to frequent secondary diagnoses of mood disorders, feeding and eating disorders, or ADHD (19). Discrepancies between caregiver reports and specialist observations are often greater for girls than for boys, with observational instruments underestimating symptom severity and delaying diagnosis and intervention (39). To address these limitations, practical countermeasures include (1) systematic integration of camouflage measures, such as the Camouflaging Autistic Traits Questionnaire (CAT-Q), into the diagnostic process (22); (2) the use of multiple informants and ecological observations to capture subtle or context-dependent behaviors (21); and (3) revision of ADOS-2 and ADI-R items with analyses of measurement invariance across sex, including probes specifically aimed at identifying camouflaging strategies (36, 40) (see **Table 1**). Implementing these strategies may reduce male-biased detection and better account for female phenotypic expressions and compensatory adaptations, ultimately improving diagnostic accuracy.

## Sociocultural determinants and psychological consequences of diagnostic delay in autistic women

Sociocultural factors play a crucial role in shaping the perception of female behavior and in influencing the recognition of neurodivergent signals. Parents, teachers, and pediatricians often hold gendered expectations—assuming that girls are naturally more relational and adaptable—which can normalize or minimize atypical behaviors, attributing them to personality traits or sensitivity rather than neurodevelopmental differences (21, 22, 43). Consequently, autistic girls often remain “invisible” within educational settings, delaying referral and diagnosis (29). Many develop early compensatory strategies, masking social difficulties through learned behaviors that demand sustained cognitive and emotional effort (22). Such camouflaging allows temporary social adaptation but comes at the cost of significant psychological strain. Supportive family contexts or small, familiar environments can further postpone the emergence of overt difficulties until adolescence or adulthood, when complex social demands—such as those encountered in university or the workplace—reveal underlying vulnerabilities (44). This prolonged mismatch between external expectations and internal experience often produces emotional exhaustion, dissociation, and psychosomatic symptoms (21, 22). The discrepancy between public functioning and private suffering fosters a loss of authenticity and self-awareness, frequently compounded by bullying, exclusion, and gendered pressures to conform (29, 43). Over time, this cycle contributes to perfectionism, rigid routines, and heightened risk for internalizing disorders such as anxiety, depression, and eating disorders (23, 24, 44). Many women continue to camouflage effectively into adulthood, with compensatory abilities often declining around menopause, when diagnosis is finally obtained after decades of misrecognition (21, 22) (see **Table 2**). Recognizing how sociocultural mechanisms interact with psychological adaptation is therefore essential for clinicians: interventions must address both the systemic biases that delay diagnosis and the internalized coping strategies that, while adaptive, contribute to emotional distress and identity fragmentation (22, 23).

**Table 2:** Camouflaging: forms, functions, and clinical implications.

Type of camouflaging	Clinical Examples	Adaptive function	Clinical consequences
Behavioral	Mimicking gestures or tone of voice	Avoid social rejection	Chronic fatigue, burnout, reduced social spontaneity, increased risk of stress-related illnesses (22, 23)
Cognitive	Prepared scripts, post-event analysis	Control of social interactions	Social anxiety, rumination, impaired decision-making, difficulty engaging in spontaneous conversation (22, 24)
Emotional	Suppression of authentic emotions	Reduce social dissonance	Depression, feelings of alienation, emotional numbness, difficulty forming genuine connections (22, 43)
Identity-related	Creation of “socially false selves”	Apparent social integration	Dissociation, loss of authenticity, identity confusion, vulnerability to long-term self-esteem issues (24, 44)

## Discussion

Early and accurate diagnosis of autism in women remains one of the most complex challenges in contemporary clinical practice. A consistent body of research indicates that standard diagnostic instruments—largely developed and validated on male samples—tend to underestimate or misinterpret the presentation of autism in females (21–23). It is worth noting that the female autistic phenotype often becomes visible only in specific transitional contexts, such as adolescence, university life, or motherhood—stages that current diagnostic protocols capture only partially (29, 44). In these contexts, compensatory mechanisms, particularly camouflaging, play a paradoxical role: they facilitate short-term adaptation to social norms but generate significant cognitive and emotional strain in the long run (22, 24). Camouflaging can manifest in several interrelated forms—behavioral, cognitive, emotional, and identity-related—each with its own adaptive purpose and potential clinical cost. Cognitive forms, including pre-rehearsed scripts or post-interaction analyses, may enhance social control while fueling anxiety and rumination (22, 43). Emotional masking often involves suppressing authentic affect to maintain social harmony, which may result in emotional numbness or depressive symptoms. Finally, identity-related masking—what some women describe as “performing a socially acceptable self”—can erode authenticity and foster dissociative experiences over time (24, 44). Thus, camouflaging can be understood as both a creative coping strategy and a psychological vulnerability, illustrating the tension between social inclusion and self-coherence. The question is not only what is observed, but also how and through which expectations observations are made. Despite recent proposals for incremental adaptations to screening tools, many approaches still rely on male-centered paradigms that fail to reflect the diversity of female neurodevelopment (36, 40). A more gender-sensitive and developmental approach is required, combining standardized tools such as the CAT-Q and GQ-ASC with multi-informant and ecological assessments (21, 22). Improving diagnostic accuracy also depends on rethinking professional training. Clinicians would benefit from structured, interdisciplinary curricula that integrate clinical neuroscience with gender studies and the sociology of neurodiversity. Such programs could help dismantle persistent stereotypes and increase sensitivity to the nuanced presentation of autism in women. A feasible pathway could follow three interconnected phases:

- Short term: integrate gender-sensitive self-report tools (e.g., CAT-Q, GQ-ASC), introduce explicit masking probes in ADOS/ADI-R assessments, and organize workshops to enhance clinicians’ awareness of female-specific features.
- Medium term: conduct systematic validation studies on measurement invariance, develop standardized gender-based training programs, and establish interdisciplinary research networks to consolidate empirical knowledge.
- Long term: promote structural reform by updating diagnostic guidelines, embedding gender-inclusive modules into medical and psychological training, and developing AI-based aids calibrated for sex- and gender-related variability.

Finally, it is crucial that autistic women participate as co-researchers and consultants in this diagnostic innovation process. Their lived experience provides insights that can bridge the gap between scientific models and real-world presentation, ultimately fostering more timely, accurate, and humane assessments.
